# Research Trends and Gaps in Construction Insulation Materials from Textile Waste and End-of-Life Wind Turbine Blades with Bio-Binders

**DOI:** 10.3390/ma19071465

**Published:** 2026-04-05

**Authors:** German Vela, António Figueiredo, Vítor Costa, Romeu Vicente

**Affiliations:** 1 Romeu da Silva Vicente (CERIS), Department of Civil Engineering, University of Aveiro, Campus Universitário de Santiago, 3810-193 Aveiro, Portugal; german@ua.pt (G.V.); romvic@ua.pt (R.V.); 2 TEMA, Center for Mechanical Technology and Automation, University of Aveiro, Campus Universitário de Santiago, 3810-193 Aveiro, Portugal; v.costa@ua.pt

**Keywords:** construction materials, insulation materials, end-of-life wind turbine blades waste, textile waste, bio-binders, circular economy

## Abstract

Waste from the wind power and textile industries poses major environmental challenges. While the textile industry is a significant global contributor to waste, producing around 92 million tons of waste annually, and greenhouse gas emissions, wind power, although one of the cleanest energy sources during operation, still generates waste and associated CO_2_ emissions, particularly associated with the end-of-life decommissioning of turbine blades. This waste can be reused, combined with bio-based binders, to reduce the construction sector’s long-term environmental impact. The present work identifies research trends and gaps in the use of these waste materials, either individually or combined, for the development of thermal and acoustic insulation solutions for the construction sector, by means of a combined bibliometric and content analysis of Scopus and Web of Science documents from 2014 to 2025. The study focuses on bibliometric indicators and reports on physical properties (thermal conductivity, density, mechanical strength, and acoustic performance) of the resulting composites, including those produced with bio-binders. Additionally, a qualitative review of life cycle assessment studies indicates that bio-based and waste-derived insulation materials can significantly reduce environmental impacts compared with conventional mineral or petrochemical insulators. Results reveal growing scientific interest in this subject, highlighting an annual publication growth of 5.09%. They emphasize the performance of natural textile fibers in thermal and acoustic insulation, the mechanical capacity of synthetic fibers, and the semi-structural potential of fiberglass composites. Meanwhile, bio-binders improve the upcycling of textile waste; however, they reveal a significant research gap in the integration of wind turbine blade waste into insulation composites. No indexed studies were found that simultaneously combine textile waste, blade-derived fibers, and bio-based binders in a single insulation system, despite projected cumulative blade waste of 43 million tons by 2050. These findings advocate hybrid innovations and standardized assessments to drive circular economy and low-carbon building solutions.

## 1. Introduction

In 2023, the construction industry was responsible for 36% of European Union (EU) energy-related Greenhouse Gas (GHG) emissions. Fossil fuels used in buildings to produce electricity for internal consumption are the main source of these emissions [1]. Furthermore, construction contributed 34% to global energy demand in 2022, revealing a considerable gap between the status and the defined target of decarbonizing the building sector by 2050 [2]. A priority objective to mitigate environmental impacts lies in the reduction of energy consumption in the construction sector, according to the EPBD recast (Energy Performance of Buildings Directive) [3] and Conference of the Parties (COP29) [4].

The improvement of the building envelope using thermal insulation is one of the key factors that can significantly impact the sustainability of the construction sector [5]. In the European market, there are mainly three types of thermal insulators: mineral and inorganic-based, which represent about 60% of the market, petroleum-based materials (particularly extruded materials), which account for 30%, and natural organic materials, which represent 10%. In this last group, expanded cork agglomerate stands out, with Portugal holding the position of the world’s largest producer and exporter of cork-based products [6]. Thus, the pursuit of creating new environmentally friendly composites for thermal insulation, incorporating waste, namely end-of-life wind turbine blades and textile waste, represents a relevant opportunity to reduce the negative impact of the construction sector.

Several researchers worldwide have carried out numerous studies on the development of biocomposites and environmentally friendly materials for use as thermal insulators, proving that these materials are competitive and either match or surpass the insulating properties of more conventional commercially available materials [7,8,9]. Although there are several works in the literature on the use of end-of-life wind turbine blades and/or textile waste for thermal insulation applications, few integrate bio-binders into the proposed solutions. Despite the small number of studies, different proposals using these materials (bio-binders) have been developed by combining them with other materials to obtain biocomposite thermal insulators, contributing to mitigating environmental problems of building operation and of the construction sector [10,11,12,13,14,15,16], thus paving the way for the valorization of waste from the wind and textile industries.

Wind turbine blades are designed to have a useful life of 20 to 25 years, after which they are decommissioned. They are generally composed of fiberglass or carbon fibers, epoxy resins, polyvinyl chloride (PVC), balsa wood, and a polyurethane coating [17]. The current trend in the wind power industry highlights the urgent need to develop effective recycling and disposal solutions due to the increasing volume of end-of-life blades. It is estimated that, by 2033, around 200,000 tons of wind turbine blades will be decommissioned each year [18]. Furthermore, the prediction of end-of-life waste flow forecasts a 2.9 million tons by 2050, while the accumulated blade waste amounts to 43 million tons per year [19,20].

In turn, the textile industry is very wasteful and produces pre-consumption waste (generated during the manufacturing process) and post-consumption waste (generated when customers discard clothes, when they are no longer in use, or in the case of fast fashion changes). There are two main types of fabrics based on the nature of the materials used: natural, such as cotton and sheep wool, and synthetic, such as polyester and nylon. A total of 92 million tons of clothing is thrown away worldwide every year, and global textile consumption is expected to be 134 million tons per year by the end of 2030 [21]. Thus, viable and sustainable approaches to textile and clothing waste management are essential. Recent studies have shown that upholstery textile fibers and artificial leather residues can be successfully incorporated into engineered panels with satisfactory mechanical performance, offering a promising route for their forward valorization [15].

Both the wind power and textile industries rely on sub-optimal end-of-life management routes for their solid waste. In the wind sector, decommissioned blades are predominantly landfilled or co-processed in cement kilns, while mechanical and chemical recycling routes are still emerging but remain limited in scale [19,22,23]. In the textile sector, most post-consumer waste is still disposed of in landfills or incinerated, and 87% of the fiber used in clothing production is ultimately treated in these facilities. This represents approximately 2 billion tons of greenhouse gas emissions, accounting for about 2% to 8% of global emissions each year [24]. Pre-consumer waste is also expected to exceed 100 million tons per year [24], with only a minor fraction being mechanically recycled or repurposed in open-loop applications [25,26]. These prevailing practices lead to resource losses and additional greenhouse gas emissions, underscoring the need for higher-value recycling pathways such as the development of insulation materials from these waste streams.

This review adopts a threefold focus on textile waste, end-of-life wind turbine blades, and bio-based binders as promising feedstocks for sustainable building thermal and acoustic insulation. While the literature reveals a substantial and growing body of research on insulation composites from recycled textile fiber, demonstrating competitive thermal and acoustic properties, no experimental studies have yet explicitly used decommissioned turbine blades as feedstock, despite their high fiberglass content. This absence constitutes the central research gap addressed in the present work and represents one of its main contributions: mapping the current state of knowledge while identifying a clear pathway for future innovation. Implementing insulation and related retrofit strategies has the potential to cut energy demand by 30% to 60% in buildings [27,28]. The review identifies trends, opportunities, and contributions that can advance the circular economy and sustainability across four interconnected sectors: wind energy, textiles, construction, and building operation [29,30].

Against this background, the review addresses three central research questions: (i) how the indexed literature on insulation materials from textile waste, fiberglass composites and bio-based binders has evolved over the last decade; (ii) what ranges of thermal, acoustic and mechanical performance are reported for these different systems; and (iii) what technological gaps and opportunities exist for combining end-of-life wind turbine blades with textile waste and bio-binders, given that no indexed studies currently explore this triple hybrid approach.

To answer these questions, the study combines bibliometric mapping with content analysis of experimental studies. The remainder of the paper is organized as follows: Section 2 details the search strategy and screening criteria; Section 3 presents the bibliometric results; Section 4 synthesizes the technical performance and circular-economy discussion; and Section 5 summarizes the key findings and future priorities.

## 2. Methods

To address the three central research questions defined in the Introduction, this study adopts a combined bibliometric and content-analysis approach. Bibliometric mapping was used to identify overall trends, geographical distribution, journal outcomes, and keyword structures across the entire corpus, while content analysis focused on quantitative experimental data to enable direct comparison of materials’ properties.

This review was based on a list of keywords, with the most relevant terms related to the field of application, in this case, “Building*” and “Construction*”. Then, terms related to specific materials of interest were added: “glass fiber*”, “fiberglass*”, “textile*”, “waste” “end-of-life”, “wind turbine*” and “blade*. Additionally, importance was given to ensure a sustainable approach to research, including terms that describe ecological characteristics: “bio”, “natural”, “vegetal”, “eco-friendly”, “binder*”, and “green”. At the same time, terms indicating insulating properties were defined: “Insulation*”, “Insulating,” and “insulat”. Furthermore, words related to conventional and advanced materials used in the construction sector, such as “concrete”, “cementitious*”, “floor*”, “aerogel” and “foam*” were excluded. This approach covered the research domains, including the field of application, materials of specific interest, and sustainable characteristics of materials.

This research was conducted in two main databases: Scopus, where the search was structured, constraining the query to the title, abstract, and keywords, resulting in 181 documents; Web of Science (WoS), a similar query returned 83 documents. Specifically, using Scopus, the following Boolean query was used: TITLE–ABS–KEY ((“Building*” OR “Construction*”) AND (“Insulation*” OR “Insulating” OR “insulat?”) AND (“textile waste” OR “glass fiber*” OR “fiberglass*” OR “Wind turbine*” OR “blade*” OR “end-of-life”) AND (“binder*” OR “bio” OR “natural” OR “vegetal” OR “eco-friendly” OR “green”) AND NOT (“concrete” OR “cementitious*” OR “floor*” OR “aerogel” OR “foam*”)). Preliminary screening of earlier records (2000–2013) showed only a few scattered publications (33 in Scopus and 13 in WoS), many of them conference proceedings without accessible full text or comparable insulation data; these items were therefore excluded to avoid adding noise and to preserve a homogeneous, data-rich corpus, and this is the main reason why the bibliometric analysis was restricted to the 2014–2025 period. After limiting the publication period to 2014–2025 and document types to articles, reviews, book chapters, and books, 136 documents remained from Scopus and 83 from WoS; 53 duplicates were removed, along with 4 documents without full-text access and 19 outside the scope, resulting in 138 documents for bibliometric and content analysis.

Different metrics were evaluated in the bibliometric analysis, namely the evolution of the number of publications, main contributions by country, most influential journals, and keywords frequency, using the R environment (RStudio 2024.04.2) and the Bibliometrix package v.4.1.3 with its Biblioshiny v.4.1.3 interface [31]. This combination of indicators allows visualizing publication trends, geographical and journal distributions, and thematic structures, and helps identify collaboration patterns and knowledge gaps, which can be considered to be opportunities for future research [32].

For the content analysis presented in Tables 1–3, a subset of 72 studies (42 on textile fiber-based composites, 17 on fiberglass-based composites, and 13 on bio-binder systems) were selected from the 138 documents identified, because these articles report quantitative experimental data on key physical properties such as thermal conductivity, density, mechanical strength, and acoustic or sound absorption performance, which are directly comparable within the predefined classifications (natural, synthetic, or mixed textile fibers for Table 1; fiberglass composites for Table 2; and bio-binder integration for Table 3). Other documents in the set, including conceptual reviews, life-cycle assessment studies, modeling papers, and works focused on related but non-insulation applications, remain valuable for contextual understanding but do not provide property data that can be harmonized in comparative tables. Therefore, they were used only to inform the bibliometric mapping and general discussion [33].

**Figure 1:**
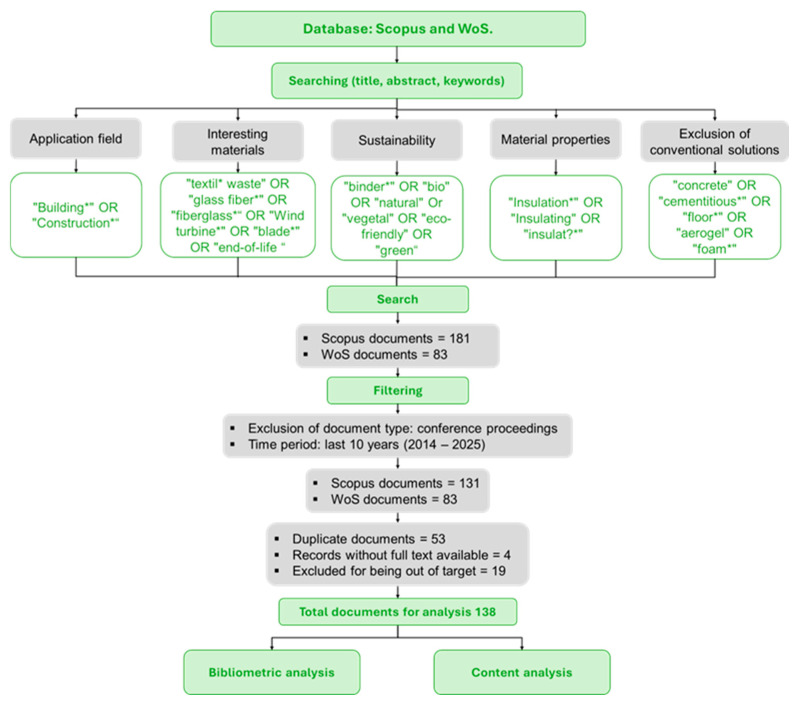
Flowchart of the literature search and selection methodology.

Figure 1 summarizes this methodology, from database searches and screening to the separation between bibliometric mapping and property-focused content analysis.

## 3. Results and Discussion

### 3.1. Research Landscape and Publication Trends

The bibliometric analysis covers the period of 2014–2025, although the first record found in the literature dates to 1985 [34], which describes the reuse of textile waste in boards, insulating panels for construction, and reinforcements for road paving [34]. Shredded textile waste was also used to recover synthetic polymers and to produce geotextiles, such as protective felts and drainage fabrics.

**Figure 2:**
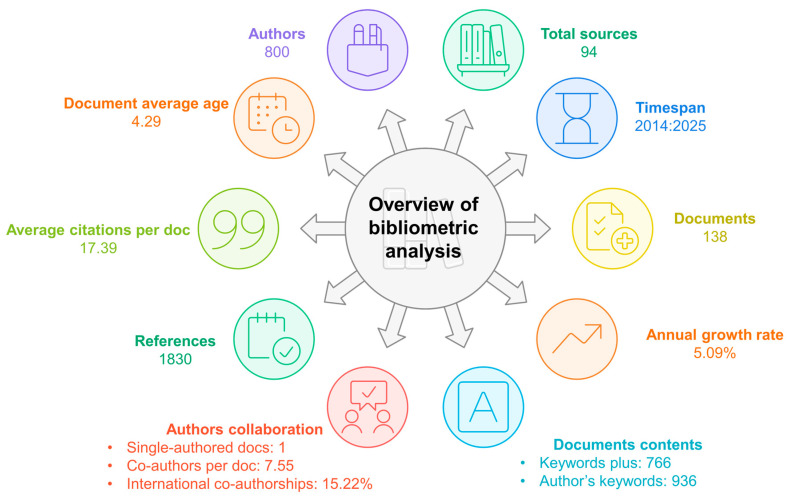
Summary of the main bibliometric information of the literature search.

Figure 2 presents an overview of the bibliometric analysis, summarizing the key characteristics of scientific production on insulation materials incorporating end-of-life wind turbine blades and textile waste with bio-binders. The analysis shows an annual growth rate of 5.09%. This relatively moderate growth rate suggests a continuous and stable increase in publication output over the past decade. The average age of the documents is 4.29 years, reflecting a balance between recent and more established contributions, and indicating the field’s capacity for continuous knowledge renewal.

In terms of impact, the average number of citations per document is 17.39, indicating notable engagement within the scientific community. Collectively, the documents cite 1830 references, highlighting a broad interdisciplinary foundation. According to authorship, 800 researchers contributed to the corpus, with only one single-authored work. On average, each document has 7.55 co-authors, and 15.22% of publications involve international collaboration. This high level of co-authorship and international collaboration clearly suggests that the development of sustainable construction materials from these waste streams requires multidisciplinary and collaborative expertise.

The thematic content of research is reflected in the use of 936 author-provided keywords and 93 sources (journals, conferences, and books), indicating a diversified dissemination landscape with peer-reviewed journals as the primary channel.

**Figure 3:**
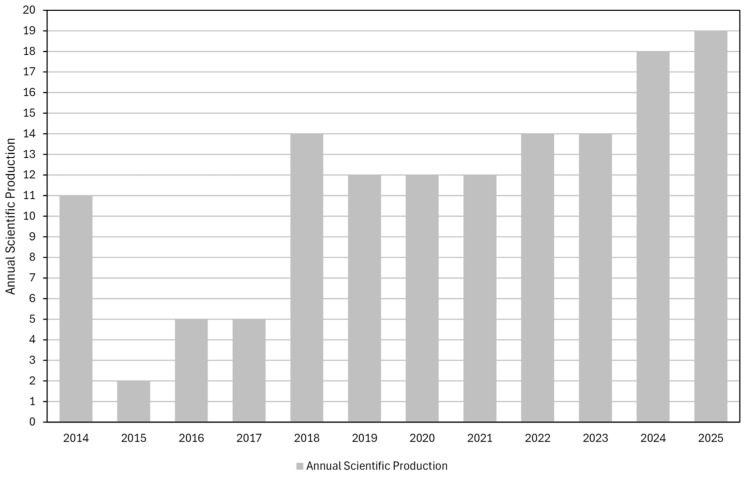
Evolution of publications and citations from 2014 to 2025.

Figure 3 shows the evolution of publications and citations. Three distinct phases are identified: low output between 2014 and 2017 (average 5.75 publications/year), recovery and growth from 2018 to 2021 (average 13 publications/year, remaining stable at 12 during the Coronavirus Disease 2019 (COVID-19) pandemic period), and accelerated expansion from 2022 to 2025 (average 16.25 publications/year). The interest in end-of-life wind turbine blades waste stems from anticipated future volumes rather than current pressure, which will strongly increase in the coming decades.

In summary, the annual scientific production and citation trends indicate a field that is still emerging and consolidating. After a period of low output, publications have increased steadily with a moderate annual growth rate and a noticeable recent acceleration, suggesting that research on insulation materials incorporating textile waste and end-of-life wind turbine blades is gaining maturity and relevance, with clear potential for further expansion and contribution to circular-economy and low-carbon construction agendas.

### 3.2. Main Contributors to Scientific Production by Country

**Figure 4:**
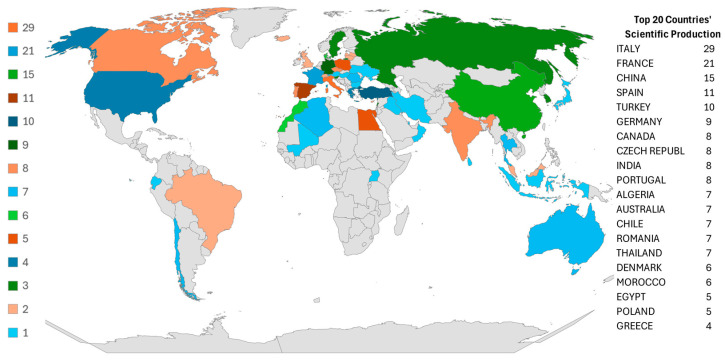
World map with the distribution of scientific production by country.

The scientific production of various countries serves as a general indicator of research relevance on the subject at the national level, enabling the identification of countries with the highest scientific or technological outputs. Figure 4 displays a color map that quantifies scientific production by country, based on the author’s affiliation.

The world map reveals a significant concentration of scientific records in specific European countries, particularly Italy (29 publications), France (21), and Spain (11), which account for approximately 45.5% of global publications. This dominance is attributed to Italy’s strong textile sector and focus on circular economy models [35]. France’s innovation in recyclable thermoplastic resins for wind energy, and Spain’s extensive wind infrastructure reaching its end of life [36]. A marked disparity in publication volume is evident among countries: for instance, publication output of Italy is over seven times higher than that of the United States of America (USA) (four publications). While nations such as China (15), Türkiye (10), and India (eight) show increasing interest in the field, their current outputs remain lower than those of leading European countries, suggesting substantial potential for growth and localized research in emerging regions.

**Figure 5:**
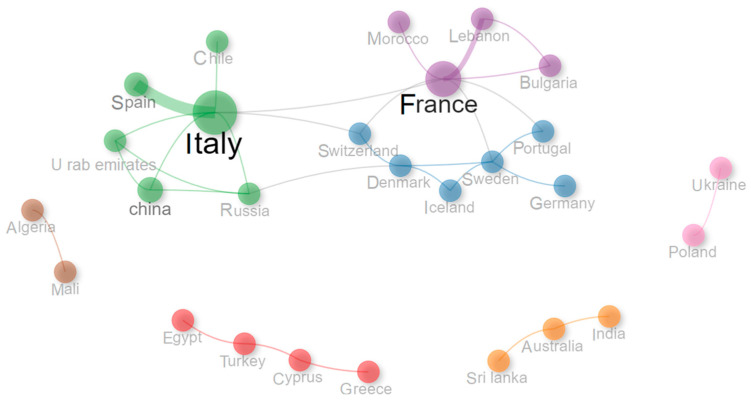
Network of scientific collaboration between countries and cluster connections.

Figure 5 gives a comprehensive picture of the collaborative international ties of countries, divided into six groups of colors.

The cluster analysis identifies collaboration communities based on connection density. This approach aims at outlining measures of Betweenness, used to highlight countries that act as bridges in the network. France shows the highest Betweenness (54.5), confirming its central role as the main connector between Europe, the Middle East, and South America. Italy follows with 46, playing a similar role in the green cluster, connecting Spain, China, Russia, and the United Arab Emirates (UAE). Both are key bridges for integration in clusters acting in the field.

In Northern Europe (blue cluster), Sweden (23.5) and Denmark (12) stand out as key brokers. Switzerland (4.5) also contributes, while others like Germany and Iceland show zero, meaning no bridge function. They remain inside the cluster but do not act as bridges within the cluster. In the Mediterranean region (red), Türkiye and Cyprus have a Betweenness of 2.0, locally but not globally. Greece and Egypt show zero, reinforcing their peripheral position in the network. Australia has 1.0 in the orange cluster, which is a modest value. India and Sri Lanka have zero (no linking) role. Algeria and Mali (brown) are isolated, with no Betweenness at all.

Overall, these geographical collaboration patterns indicate that Europe plays a central role in advancing insulation materials based on textile waste and end-of-life wind turbine blade composites. The involvement of countries with established wind and textile sectors, coupled with emerging contributions from China, India, and Türkiye, highlights important opportunities to foster new collaborative links with underrepresented regions to accelerate the global development of circular economy-based insulation solutions.

### 3.3. Scientific Landscape: Influential Journals in Sustainable Materials Research

**Figure 6:**
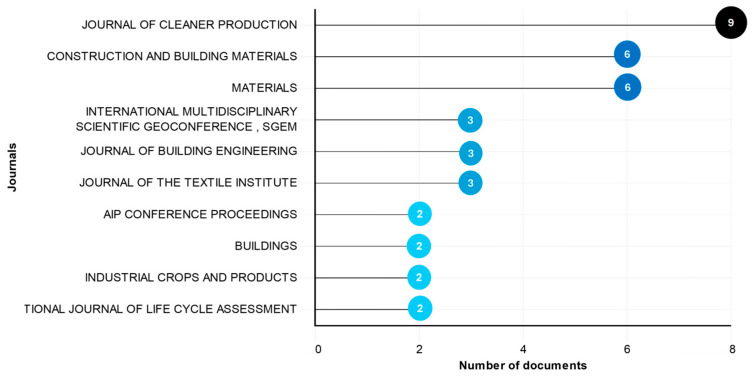
List of the top 10 journals with the largest number of publications.

As previously noted, the 138 documents analyzed in this review were published in 94 different sources, with peer-reviewed journals constituting the main dissemination channel. Figure 6 presents the 10 journals with the largest number of publications, which together provide a representative overview of where research on insulation materials from textile waste, fiberglass composites, and bio-based binders is most frequently reported.

The list is clearly dominated by materials and construction-oriented journals, particularly Journal of Cleaner Production (nine articles), Construction and Building Materials and Materials (six articles each), and Journal of Building Engineering and Journal of the Textile Institute (three articles each), indicating that the field is primarily driven by the development, characterization and application of materials for building envelopes under strong sustainability constraints. Journals such as Industrial Crops and Products and Materials Science Forum highlight the role of bio-based resources, textile engineering, and broader materials science, whereas conference proceedings (e.g., International Multidisciplinary Scientific GeoConference Surveying, Geology and Mining, Ecology and Management (SGEM) and American Institute of Physics (AIP) Conference Proceedings) show that part of the discussion still takes place in multidisciplinary fora where circular-economy solutions are debated alongside other environmentally friendly technologies.

Overall, this publication profile confirms that most current contributions concentrate on demonstrating the technical feasibility and environmental benefits of insulation materials based on textile waste and, to a lesser extent, fiberglass composites and bio-binders, within established materials and construction journals. At the same time, the absence of specialized outcomes, or recurring article series, explicitly addressing end-of-life wind turbine blades and their combination with textile waste and bio-based binders, reinforces that the proposed triple hybrid approach remains largely unexplored in the indexed literature, which motivated the third research question on technological gaps, needs, and opportunities for such systems.

### 3.4. Frequency and Relevance of Keywords

An exhaustive analysis of keywords is essential in bibliometric studies, as it strongly affects the accuracy and relevance of literature outcomes. This analysis can be performed using two complementary approaches: the author’s keyword method and the Keyword Plus method [31]. Keyword Plus terms are often more numerous and broadly descriptive than Author Keywords and can provide an effective basis for mapping the knowledge structure of a field [37,38]. In this study, the Keyword Plus methodology was therefore adopted within the Bibliometrix/Biblioshiny framework [31] to obtain a wider and more homogeneous set of descriptors across documents, thereby expanding the coverage of the main themes associated with insulation materials from textile waste and end-of-life wind turbine blades.

At the same time, this choice entails some limitations that should be acknowledged. Because Keyword Plus terms are generated from the titles of cited references, they are only available for documents indexed in Web of Science. The Scopus records do not provide an equivalent controlled set of derived keywords; the combined dataset may under-represent contributions written in languages other than English and practice-oriented work reported in non-indexed outlets. Consequently, the keyword patterns discussed in this section should be interpreted as reflecting the peer-reviewed, indexed literature rather than the full spectrum of industrial reports and non-English case studies in the field.

**Figure 7:**
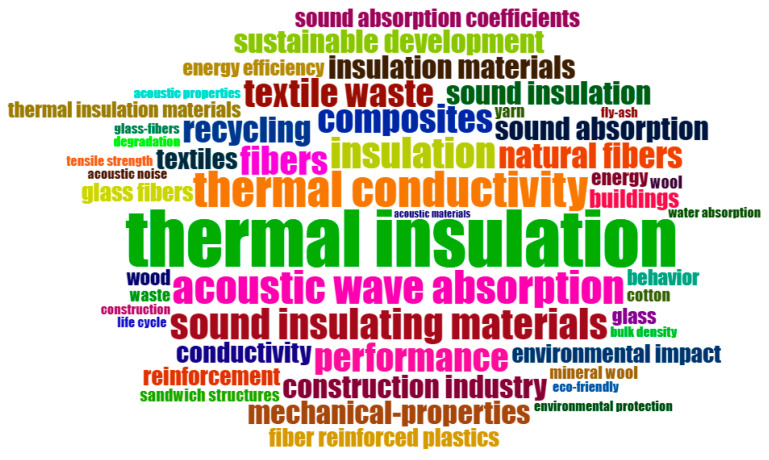
WordCloud of the most used “keywords plus” in the conducted search.

Figure 7 displays the predominant “keyword plus” cloud of this research, providing an overview of the principal subjects of study.

The keyword analysis reveals that research is mostly centered on materials and their properties, particularly emphasizing thermal and acoustic insulation. The high frequency of terms such as “thermal insulation” (22), “thermal conductivity” (14), “acoustic wave absorption” (13), “insulation” (12), “sound insulating materials” (12), and “composites” (11) suggests that studies aim at understanding and improving materials/solutions that help save energy and reduce noise in buildings, aligning with the construction industry’s trends toward comfort, innovation, and sustainability.

The construction area is a predominant topic, evidenced by the recurrent use of terms such as “buildings” (seven), “construction industry” (nine), and “construction” (four), indicating that research concentrates on the application of materials studied in construction to enhance the buildings’ sustainability, energy, and acoustic performance. The study reveals a notable interest in composite materials and natural fibers, indicated by the frequent occurrence of keywords like “composites” (11), “fibers” (11), “natural fibers” (10), and “textile waste” (11). These materials are commonly used in insulation applications due to their distinctive characteristics, including lightweight, strength, and minimal environmental impact, given their provenance.

Terminology such as “recycling” (10), “sustainable development” (nine), and “environmental impact” (seven) clearly reflect the emphasis on sustainability, meaning that research pursues to provide materials and construction solutions that reduce environmental impact and enhance energy efficiency. Additional descriptors related to mechanical properties and density highlight concerns with durability, structural performance, and lightweight design, which are essential for ensuring long-term performance in construction applications.

Nonetheless, there is a noticeable deficit in terminology such as “binder” or “bio-binder”, which exhibit low or even non-existent frequency. Similarly, explicit references to end-of-life wind turbine blades are rare in the keyword set, even though these waste streams are central to several of the reviewed studies. This indicates that blade-derived fiberglass and its recycling routes are not yet consolidated as a recognized thematic axis in the indexed literature. Consequently, while research centers mainly on the development and characterization of insulating materials for civil construction applications, the detailed treatment of bio-based matrix systems and end-of-life wind turbine blades remains an underdeveloped topic. This lack of explicit keyword signals suggests that the combined use of textile waste, blade-derived fibers, and bio-binders has not yet emerged as a distinct research theme, directly supporting the third research question on technological gaps and opportunities for developing these triple hybrid insulation systems.

The relatively high average citation rate and the concentration of research in sustainability-oriented materials journals suggest that waste-derived insulation materials are not treated as a marginal topic, but as part of the core agenda for decarbonizing the construction sector. At the same time, the contrast between the high frequency of generic terms such as “thermal insulation” and the near absence of “wind turbine blades” or “bio-binders” points to a thematic fragmentation. In practical terms, this indicates that while individual waste streams are already well studied, the community still lacks a shared vocabulary and a cohesive research framework for integrated hybrid systems. This opens a strategic opportunity for researchers and policy makers to move from single material studies towards a more coordinated approach that explicitly links blade and textile waste management with the technical and regulatory requirements of building physics.

## 4. Contents Analysis

This section presents a thematic content analysis of the 72 data-rich studies selected for Tables 1–3, organized into three main material families: textile fiber-based composites (Section 4.1), fiberglass-based composites (Section 4.2), and bio-binder-based systems (Section 4.3). For each family, the literature is discussed following a common sequence that introduces the context and typical configurations, summarizes reported thermal, acoustic, and mechanical performance, examines main limitations and application domains, and highlights emerging trends, research gaps, and future directions, which are then integrated in a broader circular-economy perspective (Section 4.4).

### 4.1. Properties of Textile Fiber-Based Composites

Table 1 summarizes data from various studies, classified by materials composition, with emphasis on composites derived from natural, synthetic, and mixed textile fibers. The table includes the main constituents (textile fiber, additional reinforcements or aggregates, binder/matrix/adhesive), and a ‘Textile waste type’ column that specifies whether the fibers originate from pre-consumer (manufacturing scraps), post-consumer (discarded garments), post-industrial (production residues), mixed waste streams, or, in a few cases, virgin or raw agricultural fibers. This explicit distinction between waste-derived and virgin sources is essential to place the reported properties in a realistic circular-economy context. The key properties include thermal conductivity (λ, in W/(m·K)), where lower values indicate better thermal insulation, particularly values below 0.07 W/(m·K), which are classified as thermal insulating materials for the construction sector [39]; density (in kg/m^3^), where reduced values allow for lightweight composites; compressive and flexural strength (in MPa), where higher values reflect greater mechanical capacity to support loads, and thus also durability; and the Noise Reduction Coefficient (NRC), an important index for evaluating the acoustic properties of materials, characterizing their effectiveness in absorbing incident sound; higher NRC values indicate greater acoustic absorption. By presenting average ranges for each category, both thermal conductivity and NRC help compare the insulation performance of the different material systems. Table 1 facilitates direct comparisons of the composites’ performance. Overall, these materials illustrate the repurpose of textile waste, in line with circular economy principles and contributing to reducing carbon emissions in the building sector (both construction and operation).

Scientifically, the waste type significantly influences fiber integrity and performance. Post-consumer waste often exhibits higher contamination levels and shorter fiber lengths due to wear and processing history, which can lead to higher thermal conductivity compared to pre-consumer sources. In contrast, post-industrial residues retain higher purity, enabling better mechanical properties and reproducibility for use in composites [33,61]. This classification aids in assessing sustainability, as waste-derived fibers reduce virgin resource demand but require optimized processing to mitigate variability in properties. For example, the fragmented fibers in post-consumer waste enhance open porosity, leading to lower densities (e.g., 20–50 kg/m^3^ in wool/cotton/polyester mixes), low thermal conductivities (λ = 0.039–0.049 W/(m·K)) and high acoustic absorption (NRC up to 0.83) via increased air entrapment that disrupts heat conduction and sound propagation; however, this degradation compromises mechanical strength (<0.3 MPa) by introducing defects.

Conversely, pre-consumer and post-industrial types promote denser packing (e.g., 240–340 kg/m^3^ in flax composites) and higher flexural strengths (up to 3.35 MPa) through improved fiber-matrix bonding, though at the potential cost of reduced porosity and slightly higher thermal conductivity. This interconnection highlights trade-offs in circular economy applications, where enhanced thermal and acoustic performance is usually combined with lower mechanical capacity and vice versa.

Based on the 42 unique studies compiled in Table 1, approximately 60% of the composites are predominantly based on natural textile fibers, 40% on synthetic or mixed fibers, and around 26% combine waste-derived and virgin textile components, confirming the predominance of natural-fiber-based solutions in insulation research.

Regarding thermal performance, natural fibers (sheep wool, flax, cotton, hemp) exhibit the lowest thermal conductivity values (0.029–0.089 W/(m·K)), positioning them as thermal insulators. For example, flax composites with biologically derived resins achieve values of 0.029 W/(m·K), comparable to commercial thermal insulation materials like mineral wool (0.032–0.040 W/(m·K)) [74]. This thermal performance can be attributed, in part, to the characteristic microstructure of many natural fibers, which often feature porous or honeycomb-like cross-sections with numerous air-filled micro-voids trapped inside [75]. These porosities reduce conduction and convection heat transfer, thereby favoring thermal insulation.

In contrast, composites incorporating dense mineral matrices (Portland cement, gypsum, sand) with different types of textile fibers show significantly higher thermal conductivities (0.83–1.17 W/(m·K)), approaching the behavior of conventional concrete (1.4–2.0 W/(m·K)) [74]. This suggests an inverse relationship between structural functionality and thermal insulation characteristics: the higher the mineral content, the poorer the thermal insulation performance.

On the other hand, composites based on synthetic textile waste (denim) mixed with some agricultural residues present an intermediate range of thermal conductivity (0.076–1.14 W/(m·K)). This wide range reflects the strong influence of the ratio between organic and inorganic matrix: the higher the content of lightweight and porous organic components, the lower the thermal conductivity. These results indicate that the strategic combination of synthetic fibers with natural or agricultural materials can offer a balance between structural and thermal insulation, opening new avenues of research for the development of sustainable construction materials with improved insulation properties.

Regarding density, composites with natural fibers present the lowest values between 21–340 kg/m^3^, as in the case of hemp/flax, which favors non-structural applications. Mixtures with EPS or cement increase density to >1000 kg/m^3^, which compromises transportability or implies on-site manufacturing.

Meanwhile, the lowest densities (20–83 kg/m^3^) correspond to flax/hemp composites with sodium silicate, highlighting the composite with mycelium as the lowest density recorded in the study (0.72 kg/m^3^). At the other extreme, cotton/flax composites with cement reach quasi-structural levels (1900 kg/m^3^). This wide range allows designing materials depending on the need, classifiable between non-structural thermal insulators (<200 kg/m^3^) and semi-structural or cladding materials (>600 kg/m^3^) [76].

The less dense materials have the advantage of being prefabricated, unlike the denser ones, which compromises their transportability or implies their manufacturing be carried out in situ; in particular, it stands out that the low density of composites with natural fibers and biopolymeric matrices (PLA, alginate, chitosan) is directly correlated with the lightweight and porous nature of these materials, which significantly contributes to reduce the composite overall density. In general, lower density and higher open porosity correlate with better thermal insulation and sound absorption, whereas denser systems prioritize mechanical resistance at the expense of insulating performance.

In terms of mechanical properties, composites with synthetic fibers, especially when combined with cementitious or synthetic resin matrices such as gypsum, cement, or PLA, offer the best performances, with compressive strengths reaching up to 11 MPa [69] and flexural strengths up to 13 MPa [73], making them suitable for load-bearing panels. Hybrid or mixed-fiber materials present an intermediate balance (1.5–3 MPa in compression), although their high variability suggests certain fragility and critical dependence on manufacturing conditions.

In contrast, composites based on natural fibers with biopolymeric matrices or mechanical bonds (such as gum arabic, alginate, or carding processes) exhibit very low strengths (<0.3 MPa), limiting their use to non-structural applications, mainly as thermal or acoustic insulators. In general, the type of fiber and, above all, the nature of the matrix are determining factors of mechanical behavior: mineral or synthetic matrices confer rigidity and strength, while natural or porous matrices prioritize lightness and insulation at the expense of limited structural capacity.

Finally, the best acoustic performance (NRC) is observed in natural fibers, with values ranging between 0.40 and 0.83 in sheep wool and cotton, due to their fibrous porosity. Synthetic and mixed fibers achieve an NRC of 0.15–0.59, suitable for medium noise, although inferior to pure naturals. The highest NRCs are associated with low density (20–200 kg/m^3^) and porous matrices such as mycelium, gum arabic, or natural rubber, in the absence of mineral sealing phases. Highlighted examples include cotton/polyester with natural rubber (NRC = 0.5–0.7) [60], sheep wool (NRC = 0.40–0.65) [8,33,40,41,42,43,44], and cotton with mycelium (NRC = 0.83) [53]. In contrast, materials with cement or gypsum present NRC ≤ 0.5, even with fibers, due to pore sealing by the mineral matrix. Overall, acoustic absorption critically depends on open porosity, low bulk density, and the absence of dense coatings, clearly favoring lightweight and organic composites over mineral-dominated systems.

In summary, Table 1 reveals the potential of textile fibers in building insulation. Natural fibers balance thermal and acoustic insulation (λ < 0.06 W/(m·K), NRC up to 0.83), and synthetic fibers prioritize mechanical strength (compression up to 11 MPa; flexural up to 13 MPa). Hybrid and mixed fibers offer versatile applications; in general, all applications vary according to the type of matrix (biological or mineral), which influences properties and purposes, from lightness to durability, requiring optimizations to reduce inconsistencies. This view highlights the opportunities and positions of these composites as sustainable construction materials for buildings.

When compared with fiberglass-based composites discussed in Section 4.2, these textile fiber systems generally prioritize low thermal conductivity, low density, and high sound absorption over structural capacity, making them particularly suitable for non-structural insulation layers and acoustic linings. In several cases, the use of bio-based binders or biopolymeric matrices in textile composites further enhances their environmental profile, but often at the expense of mechanical strength. The performance patterns observed in Table 1 provide a complementary counterpart to the semi-structural potential of fiberglass solutions and to the bio-binder-based systems reviewed in Section 4.3. Ultimately, this analysis of 42 unique studies confirms a consistent trend: while natural fibers excel in thermal and acoustic insulation, synthetic systems prioritize mechanical strength, with the matrix type remaining the dominant variable. These findings highlight the distinct but synergistic roles that textile and fiberglass wastes can play, reinforcing the need for hybrid approaches and careful matrix design to develop the next generation of sustainable insulation materials.

### 4.2. Properties of Fiberglass-Based Composites

Table 2 compiles data of composites derived from fiberglass (including recycled ones), with various reinforcements such as natural fibers, tire residues, crushed aggregates, and binders like Portland cement, epoxy, and gypsum. These materials are intended for thermal and acoustic insulation, and structural applications in construction, promoting sustainability through recycling. However, it should be emphasized that although several studies listed in Table 2 involve composites containing fiberglass or generic fiber-reinforced polymer (FRP) waste, none explicitly use end-of-life wind turbine blades as a feedstock. This confirms that current research on fiberglass-based insulation materials remains largely decoupled from the specific challenge of wind turbine blade recycling.

Based on the 17 studies summarized in Table 2, approximately 70% of fiberglass composites utilize epoxy or polymer matrices for enhanced mechanical strength, 20% incorporate cementitious binders for structural applications, and 10% explore biocompatible alternatives, highlighting their semi-structural potential but limited integration with waste from wind turbine blades.

Thermal conductivity (λ) spans over a wide range, from 0.030 W/(m·K) (recycled tire rubber in an inorganic matrix) to 0.83 W/(m·K) (FRP grids in mortar/geopolymer). This large spectrum reflects two clearly identifiable groups: those with λ < 0.07 W/(m·K), such as recycled tire rubber, Tetra Pak/textile waste with polypropylene, and bicomponent polyester fibers, positioning them in the range of commercial thermal insulators (mineral wool: λ ≈ 0.029–0.042 W/(m·K)). Those with intermediate or high thermal conductivity (λ > 0.12 W/(m·K)), such as snail shell/RPP, date palm fiber/Unsaturated Polyester Resin (UPR), and FRP in mortar, are associated with dense matrices (thermoset resins, mortars) or high fractions of mineral loading, increasing conduction heat transfer. Overall, the best insulation performance is associated with low-density, polymer-rich systems with high rubber, cork, or textile fractions, whereas fiberglass-reinforced mortars and geopolymers tend to behave as structural materials with comparatively poor thermal and acoustic insulation performances.

Density of the analyzed composite materials spans a wide range: from 210 kg/m^3^ in systems based on bicomponent polyester fibers to reported values of 1582–1900 kg/m^3^ in date palm fiber composites with unsaturated polyester resin, allowing them to be classified into three functional categories according to density. Ultralight materials (ρ < 400 kg/m^3^), such as bicomponent polyester fibers (210–400 kg/m^3^) and flax fiber/agglomerated cork (240–340 kg/m^3^), are mainly suited for non-structural insulation or lining applications, potentially including systems with foams or highly porous structures. Light-to-medium density materials (400 ≤ ρ ≤ 1000 kg/m^3^) include polyester resin/PVC sheets (445.5–604.2 kg/m^3^), crushed mineral aggregates with glass fiber (low end: 467–1000 kg/m^3^), and composites with Tetra Pak/polypropylene, balancing lightness and cohesion for interior lining panels or semi-rigid insulation. Dense materials (ρ > 1000 kg/m^3^), such as gypsum reinforced with tissue paper and natural rubber latex (density up to 1136.3 kg/m^3^), flax fiber with PLA (1240–1450 kg/m^3^), crushed aggregates (up to 1807 kg/m^3^), and date palm fiber composites (1582–1900 kg/m^3^), oriented toward semi-structural or lightweight structural applications, where high density contributes to rigidity and strength.

The mechanical strength of composites varies by orders of magnitude, reflecting the decisive influence of the matrix type, nature of the reinforcement, and quality of the fiber-matrix interface. Compressive strength shows the highest values in composites with reinforced mineral matrices: mortar with FRP grid (5.9–9.1 MPa), mortar with tire rubber (1.87–6.86 MPa), and cement with aggregates and fiberglass (0.92–8.54 MPa). These ranges approximate the ≥2.5 MPa required for lightweight cellular concrete blocks European Norm (EN 771-4) [88]. In contrast, gypsum with paper/rubber shows lower strengths (0.197–1.980 MPa), limiting its use to non-critical applications from the mechanical viewpoint. Regarding flexural strength, the potential of natural fibers in polymeric matrices stands out: date palm fiber/UPR (14.71–105.71 MPa), a noticeable value comparable to conventional fiberglass composites, attributable to a high reinforcement fraction and good adhesion under controlled manufacturing conditions; snail shell/RPP (30.16–34.56 MPa), with high rigidity but limited ductility; and gypsum with paper/rubber (0.01–0.90 MPa), very low and typical of brittle materials without continuous reinforcement. Altogether, these results show that fiberglass-based composites in Table 2 are generally designed to favor mechanical performance over thermal insulation, in contrast with many textile-based systems.

The Noise Reduction Coefficient (NRC) is reported in a limited manner in Table 2, only in four cases, highlighting the need for more standardized data to evaluate the acoustic performance of fiberglass composites. Gypsum with tissue paper and natural rubber shows a low NRC (0.13–0.39), due to its dense mineral matrix that limits porosity; similarly, cork with flax and epoxy reaches only 0.15–0.18, due to the sealing action of resin. In contrast, brick/limestone aggregates with fiberglass and cement achieve a moderate NRC (0.23–0.34), thanks to their hybrid structure. The standout case is centrifugal glass wool in gypsum, with an NRC of 0.77, preserving its open fibrous acoustic absorption in hybrids, unlike encapsulated short fibers. This last case is key: it demonstrates that the porous architecture of recycled fiberglass (as wool) can preserve its acoustic functionality even in hybrid systems, unlike short fibers or particles encapsulated in resins.

Finally, the role of recycled fiberglass in Table 2 composites is limited to three entries marked with ✓, highlighting its primary application as structural reinforcement rather than thermal or acoustic insulator, in contrast to its continuous fibrous variant as glass wool. For example, the FRP grid in mortar/geopolymer [78] improves compressive strength (5.9–9.1 MPa) and flexural strength (2.2–3.1 MPa), although it raises thermal conductivity (0.234–0.83 W/(m·K)), compromising thermal insulation; the crushed mineral aggregates (brick/limestone) with fiberglass and white Portland cement [80] mechanically reinforce (compression 0.92–8.54 MPa; flexion 0.59–3.06 MPa) and offer a moderate NRC (0.23–0.34), but lack data on λ. Finally, polyester resin/PVC sheets [10] present intermediate-high λ (0.133–0.190 W/(m·K)) and density (445.5–604.2 kg/m^3^). This trend suggests that recycled fiberglass prioritizes durability and stiffness in semi-structural uses, reserving its best thermal and acoustic insulation behavior for unprocessed forms such as glass wool, which invites further hybrid research to enhance its sustainable versatility.

The content analysis of these 17 fiberglass studies shows that recycled fibers are predominantly used for semi-structural reinforcement rather than insulation. This highlights a clear divergence from textile-based systems and underscores the untapped potential of blade-derived fibers; although the intrinsic performance of fiberglass is well documented, the specific challenges associated with processing large-scale wind turbine blade waste into suitable fillers for low-density insulation products have not yet been addressed in the peer-reviewed literature.

In summary, Table 2 illustrates the versatile potential of fiberglass composites in sustainable construction, allowing combinations of thermal and acoustic insulation with reinforcement according to the matrix and reinforcements employed. This compilation positions these materials in the state-of-the-art, promoting upcycling of waste to reduce environmental impacts. Future studies could focus on bio-mineral hybrids and on the integration of blade-derived fiberglass into low-density matrices, in order to exploit both their mechanical capacity and their insulating potential, boosting their commercial adoption.

Compared with the textile fiber-based composites presented in Section 4.1, fiberglass composites tend to prioritize higher mechanical strength and semi-structural performance, often at the cost of higher density and less favorable thermal and acoustic insulation. While the datasets analyzed in this review do not report experimental systems combining textile and fiberglass in a single composite, the contrasted property profiles observed in Table 1 and Table 2 suggest that such hybrid solutions could exploit the superior insulating and sound-absorbing behavior of textile fibers together with the load-bearing and stiffness contributions of fiberglass-based elements. This complementary behavior is particularly relevant when considered alongside the bio-binder-based systems discussed in Section 4.3, which offer additional opportunities to align these material strategies with circular-economy and low-carbon construction objectives.

### 4.3. Research Articles Employing Bio-Binders, and Challenges

Within this review, bio-binders are treated as a key component of insulation composites made from textile waste and end-of-life wind turbine blade fiber, as they significantly affect both environmental performance and upcycling feasibility. Table 3 presents research on bio-binder-based systems combined with this waste, focusing on those developed for thermal or acoustic insulation, or showing relevant properties such as low thermal conductivity, high porosity, or good fiber compatibility. It includes details on the materials used and their key performance indicators of thermal conductivity, density, and compressive strength, highlighting the role of bio-binders in enabling the integration of these waste streams into building insulation. The objective of the analysis is to identify the types of bio-binders used in different studies and to evaluate the integration of end-of-life wind turbine blades and/or textile waste. Analysis of the documents led to the conclusion that only solutions containing textile waste use bio-binders. Thus, no works were found in the literature combining bio-binders with end-of-life wind turbine blades, highlighting a clear research need and opportunity.

Various combinations of natural and synthetic materials were observed, mainly for thermal and acoustic insulation and structural purposes. The most used natural materials include plant fibers and textile waste, promoting sustainability and functional performance. From the studies summarized in Table 3, about 46% of the identified bio-binder applications rely on polysaccharide-based systems (e.g., starch, alginate, chitosan), 31% on polyester- or polylactide-type bio-based polymers, and the remaining 23% on plant-derived oils or latex binders. Notably, 100% of these uses are associated with textile waste feedstocks, with none combining bio-binders with end-of-life wind turbine blades composites.

Among the plant fibers used, such as hemp, jute, coconut fibers, and bamboo particles, hemp and sheep wool stood out, presenting the best properties. For example, hemp and sheep wool were used in combination with polylactic acid (PLA), providing good thermal insulation and acoustic absorption, with thermal conductivities ranging from 0.034 to 0.045 W/(m·K) [33]. Jute fibers and biaxial flax fibers, together with Karanja oil-based epoxy resins, contributed to improving the mechanical strength of the resulting composites.

While textile waste includes both natural and synthetic fibers, such as cotton, polyester, and denim waste, for the production of biocomposites, these represent a meaningful option in the reuse of post-consumer materials. Cotton and polyester are bonded with natural latex, exhibiting good flexural strength and acoustic properties, while denim waste, in combination with corn starch, is used for the development of acoustic panels.

However, biocomposites made from natural and synthetic materials, like basalt fibers and beech sawdust mixed with PLA, had the highest flexural strengths, reaching up to 79 MPa [89].

Bio-binders used in the studies vary in terms of frequency of use, innovation, and performance concerning specific properties. Numerous studies have extensively used calcium alginate and PLA. Researchers used calcium alginate with cotton and viscose fibers due to its ability to withstand heat and sparks [51]. Conversely, researchers mixed PLA with beech sawdust, basalt fibers, hemp fibers, and sheep wool waste. Chitosan comes from crustacean waste, and from the literature, it could be concluded that it works well when combined with miscanthus giganteus, rice husk, textile waste, and merino wool fibers, giving them good mechanical and thermal properties. Using corn starch to bond denim textile waste and hemp straw significantly enhances the mechanical and acoustic properties of composites [71]. Additionally, arabic natural glue was employed with flax fiber waste, achieving a low thermal conductivity of 0.029–0.325 W/(m·K) and densities from 25 to 650 kg/m^3^, as characterized by modified guarded hot box, hot and cold room methods, and steady-state techniques [50]. These bio-binders enable lightweight composites with competitive thermal insulation and, in several cases, enhanced mechanical and acoustic performance.

On the other hand, new research directions are emerging, such as the use of mycelium and other materials derived from microorganisms. Mycelium demonstrated the ability to bond bamboo particles, forming a biocomposite with thermal insulation properties and good mechanical strength. The ability of mycelium to grow and bond with different materials, forming lightweight and durable composites, represents an innovative and sustainable approach to materials manufacturing [90].

Regarding characterization techniques, a broad set of methods has been used to evaluate the properties of composite materials. Among the most used are Scanning Electron Microscopy (SEM), essential for analyzing the microstructure of materials, and thermal analysis, including DSC, DTA-TG, and TGA, used to evaluate thermal stability and decomposition properties. Fourier Transform Infrared Spectroscopy (FTIR) is frequently used to identify functional groups and analyze the chemical composition of materials. Moreover, mechanical tests (tension, bending, and compression) were widely conducted to determine the strength of composites. Measurement of thermal conductivity (λ) is crucial for evaluating the thermal insulation performance of solutions, and water absorption analysis is of paramount importance regarding structural integrity and durability of the developed solutions. These techniques allow understanding and quantification of physical, chemical, mechanical, thermal, and acoustic properties of composites, enabling a comprehensive assessment of their performance.

When interpreted together with the textile and fiberglass composites discussed in Section 4.1 and Section 4.2, these bio-binder-based systems emerge as a key lever to improve the environmental profile of insulation materials while maintaining acceptable functional performance. In textile fiber composites, bio-binders can reinforce the already favorable thermal and acoustic behavior, although often at the cost of reduced mechanical strength, whereas in fiberglass or hybrid composites, they offer opportunities to partially offset the embodied impacts of mineral or petrochemical matrices. The literature reviewed did not identify studies that simultaneously combine textile waste, end-of-life wind turbine blade fibers, and bio-based binders, revealing a clear research gap and suggesting that future work should explore multi-waste and bio-binder hybrid formulations, supported by systematic comparisons of thermal, acoustic, mechanical, and durability properties.

Overall, the content analysis of textile fiber-based composites, fiberglass-based composites, and bio-binder systems shows that each material family contributes distinct but complementary strengths to sustainable insulation: textile fibers offer good thermal and acoustic performance at low density, fiberglass composites provide higher mechanical and semi-structural capacity, and bio-binders improve the environmental profile of both, albeit sometimes with trade-offs in strength or processability. Although the existing literature typically addresses these waste streams and binder systems separately, the trends identified in Section 4.1, Section 4.2 and Section 4.3 clearly point to the potential of future hybrid solutions that combine textile waste, end-of-life wind turbine blade composites, and bio-based binders. These findings show that, while bio-binders are often studied in broader composite contexts, their most promising role lies in enabling low-impact matrices for textile- and fiberglass-based insulation systems. Notably, no current studies explicitly use decommissioned wind turbine blades as a feedstock for insulation, indicating that blade-derived fiberglass remains an unrealized opportunity rather than an established solution, and highlighting a key research gap at the intersection of waste management, recycling technology, and building physics.

The lack of research integrating end-of-life wind turbine blade fibers into insulation applications with bio-binders is driven by three primary barriers. First, technical recovery is hindered by the complex multilayered composition of blades, which typically combines epoxy resins and reinforcement fibers with balsa wood or PVC foam cores. These materials require difficult separation processes to obtain homogeneous fiber streams, often resulting in degradation of fiber length and structural integrity. Second, compatibility issues arise because residual resins or contaminants (such as char) on recovered fibers impair their ability to bond effectively with new bio-based matrices. Finally, economic and scalability constraints remain a significant hurdle, as advanced recycling routes such as pyrolysis and solvolysis have not yet achieved cost parity with conventional disposal methods. Landfilling in the United States costs approximately $60 per ton; specialized composite waste handling in Europe can range from €120 to €300 per ton, making advanced recycling less attractive than landfilling or cement-kiln co-processing [30,91].

To bridge this gap, future research should prioritize: (i) hybrid mechanical–chemical pre-treatments to obtain clean fibers longer than 5 mm with minimal dust; (ii) development of tailored bio-binder formulations (e.g., chitosan or PLA modified with coupling agents) for improved fiber-matrix adhesion; and (iii) pilot-scale demonstration of hybrid textile–blade–bio-binder composites under realistic building conditions (hygrothermal cycling, fire performance, and long-term durability). Such targeted studies would transform the current research gap into a practical pathway for high-value upcycling.

### 4.4. Circular Economy Implications, Gaps, and Future Directions

Building on the performance trends discussed in Section 4.1, Section 4.2 and Section 4.3, this section integrates those findings in a circular-economy perspective and highlights how hybrid systems, combining textile fibers, blade-derived fiberglass, and bio-binders, can address the technological gaps identified in research question (iii).

From a circular economic perspective, the management of end-of-life wind turbine blades is an escalating challenge, as global waste accumulation is projected to reach 43 million tons by 2050 [29,36,91]. Currently, landfilling remains the most common disposal method for these blades in the United States, resulting in the loss of valuable composite materials and posing environmental risks, such as the potential release of methane and other volatile organic compounds during biodegradation [36,91]. In parallel, the global textile industry faces significant sustainability hurdles, with recycling rates typically remaining around 15% [92]. Nevertheless, textile waste already serves as a viable feedstock for producing thermal and acoustic insulations in the construction industry [92]. Treating blade and textile wastes as complementary resources, leveraging the structural contribution of fiberglass from blades together with the thermal and acoustic performance of textile fibers, opens the way for higher-value construction products, such as hybrid insulation panels in which blade-derived fiberglass, textile waste, and bio-binders are combined to balance mechanical strength with thermal and acoustic performance [91,93,94]. These initiatives help align the wind, textile, and construction industries with the European Green Deal’s goal of 32% renewable energy by 2030, as well as specific national regulations like the French decree that mandates a 55% recycling rate for wind rotor mass by 2025 [30].

Current practices for blade waste emphasize mechanical shredding or co-processing in cement kilns, which typically result in low-value recycles (powders) or complete fiber destruction, where glass only serves as a mineral source for clinker [91,93]. Emerging technologies offer greater retention of material properties. Pyrolysis at 400–600 °C recovers 75–77% of fibers alongside energy-rich oils and gases, though fiberglass strength may decrease significantly [93,94]. Chemical solvolysis achieves >90% resin dissolution, yielding fibers with high retained strength and near-virgin surface quality [93]. However, these materials are often relegated to filler applications rather than structural building products [93]. Therefore, there is a critical need to standardize processing strategies that deliver controlled fiber morphology, and to systematically evaluate the compatibility of recovered fiberglass with bio-binders and textile reinforcements, so that blade-derived composites can move beyond generic filler applications and be engineered specifically as components of hybrid insulation systems [94].

From a circular economic standpoint, the asymmetry observed in the literature (characterized by numerous experimental studies on textile-based insulation systems versus a complete absence of indexed insulation composites explicitly using end-of-life wind turbine blades) should be interpreted as a well-defined research gap rather than as evidence of technical infeasibility. Addressing this gap is essential to scale these materials through robust regulatory and market enablers. Systemic instruments such as Extended Producer Responsibility (EPR) schemes for the wind and textile sectors, coupled with green public procurement criteria, are essential to incentivize the diversion of waste toward high-value applications [93]. Pioneer regulations, such as the French decree of 2020, already set that 35% of rotor mass must be reused or recycled by 2022, a target that escalates to 55% by 2025 [30]. Establishing standardized certification pathways, harmonized regulations for composite waste, and digital product passports will be critical to bridge existing traceability gaps [30,93]. In this context, policies that prioritize high-value recycling routes can help direct both textile and wind turbine blade-derived fibers, in combination with bio-binders, towards insulation products that deliver tangible environmental benefits in the building sector, rather than towards low-value or disposal options [94].

The synthesis of these experimental studies reveals a functional complementarity that serves as a design guide for the industry. The data indicate that textile-fiber composites operate primarily in the high-porosity domain, making them suitable for specialized thermal and acoustic linings, but their low mechanical strength means they cannot be used as self-supporting elements. Conversely, fiberglass composites shift the performance envelope toward structural durability, suggesting that their natural role is to act as the mechanical skeleton of hybrid panels rather than as the primary insulator.

A critical finding is that bio-binders act as the sustainable matrix needed to bond phases, although they often require a trade-off in stiffness compared with synthetic resins. In practical terms, this implies that the most viable route is not a simple material substitution, but the engineering of triple-hybrid architectures that use blade-derived fiberglass for structural strength, textile waste for porosity (thermal or acoustic insulation), and bio-binders for low-carbon cohesion. This mapped synergy allows researchers and practitioners to prioritize hybrid configurations that can realistically meet building-code requirements, turning the identified research gap into an opportune pathway for high-value upcycling in the construction sector.

### 4.5. Environmental and Practical Insights from LCA Studies

Recent life cycle assessment studies on bio-based and waste-derived insulation systems indicate that these materials generally achieve better environmental performance than conventional mineral or petrochemical solutions [12,95,96,97]. Bio-based insulations based on miscanthus, hemp, flax, wood fibers, and cork consistently exhibit reduced primary energy demand and lower abiotic resource depletion compared to mineral wool, EPS, or polyurethane, while recycled PET panels and other waste-based systems can decrease the environmental impacts of standard wall assemblies by roughly 13–45% [12,95,98]. Building-scale LCAs further show that increasing envelope insulation can reduce operational heating and cooling loads by up to about 60%, lowering whole-building greenhouse gas emissions by around 40%, so that the additional embodied impacts of higher-performance or thicker insulation layers are typically compensated during the use phase [95,99,100]. At the same time, the literature emphasizes the role of biogenic carbon storage in wood, straw, and cork-based products, which can even lead to negative cradle-to-gate global warming potentials in the product stage, partially offsetting impacts from other life cycle phases [97,98]. However, these environmental benefits are conditioned by several critical aspects such as the need for synthetic additives and fire retardants, increased thickness or density to reach target U-values, sensitivity to moisture and biological degradation, and, in particular, fire performance and smoke emissions [12,96,98,101]. Inadequate durability or protection may lead to premature replacements, effectively doubling the embodied impacts of the building elements [98].

Overall, the reviewed literature suggests that for textile and blade-derived systems, environmental performance will depend less on matching the footprint of conventional mineral wool per kilogram and more on optimizing the whole assembly over its service life. From the research and industrial perspectives, the priority is to couple the development of hybrid textile, fiberglass, and bio-binder composites with dedicated LCAs that explicitly account for processing routes, fire-protection strategies, and realistic replacement scenarios [12,95,96,98]. This approach will allow these materials to be positioned transparently as alternatives to the incumbent insulation solutions in design and policy decisions, aiming to transition from simple cradle-to-gate metrics to a comprehensive life-cycle value proposition.

## 5. Conclusions

This bibliometric and content study, based on the analysis of 138 documents from 2014 to 2025, reveals a maturing yet moderately growing research field (5.09% annual growth rate) in developing sustainable thermal and acoustic insulation materials for construction, leveraging end-of-life wind turbine blades and textile waste. Europe, led by Italy (29 publications), France (21), and Spain (11), dominates research output (45.5% of global publications), with strong international collaborations centered in France and Italy as key network bridges. Publications emphasize materials characterization, thermal/acoustic properties, and sustainability, as evidenced by frequent keywords like “thermal insulation” (22 occurrences), “composites” (11), and “recycling” (10), disseminated primarily in sustainability-focused journals such as Journal of Cleaner Production (9 articles).

Contents analysis of textile fiber-based composites underscores their potential for circular economy applications: natural fibers (e.g., sheep wool, flax, cotton, hemp) deliver low thermal conductivity (0.029–0.089 W/(m·K) and acoustic insulation (NRC up to 0.83), attributed to porous microstructures, while synthetics (e.g., denim, polyester) allow improved mechanical strength (compression strength up to 11 MPa, and flexural strength up to 13 MPa). Hybrids offer versatility but exhibit variability tied to matrix type (bio vs. mineral), with low-density options (<200 kg/m^3^) suiting non-structural prefabrication and denser ones (>1000 kg/m^3^) enabling semi-structural uses. Similarly, fiberglass composites present broad functionality, with low-*λ* groups (<0.07 W/(m·K), e.g., tire rubber hybrids) rivaling commercial thermal insulators, though recycled variants favoring reinforcement over insulation, highlighting trade-offs in density (210–1900 kg/m^3^) and mechanical strength (flexural up to 105.71 MPa in date palm/UPR).

Bio-binder integration is limited to textile waste solutions (e.g., PLA with hemp/sheep wool: *λ* = 0.034–0.045 W/(m·K); NRC = 0.60–0.65), enhancing thermal/mechanical properties via natural agents like calcium alginate, chitosan, corn starch, and mycelium, while no studies combine them with wind turbine blade materials. This reveals and highlights a critical gap amid the projected cumulative blade waste of 43 million tons by 2050.

Beyond the identification of research gaps, this review establishes a strategic framework for the next generation of circular economy construction materials. The evidence suggests that technical and environmental viability depends on a synergistic ‘triple-hybrid’ approach: utilizing the high porosity of textile waste for thermal and acoustic insulation performance, harnessing the mechanical stiffness of wind turbine fiberglass for structural strength, and incorporating bio-based binders for low embodied carbon. Overall, recycled textile fibers already underpin a wide range of technically viable thermal and acoustic insulation composites, while wind turbine blade-derived fiberglass remains an unrealized opportunity rather than an established solution, and bio-binders offer a promising route to reduce the environmental footprint of both material families. For industry and regulators, the practical implication is clear: instead of seeking a direct one-to-one replacement for mineral wool, efforts should be directed toward engineering composite architectures that enhance the whole-building life cycle. Future work should prioritize multi-criteria assessments that jointly address performance, durability, cost, and life cycle impacts, supported by dedicated cradle-to-grave assessments that explicitly account for fire safety and realistic replacement scenarios, so that these waste-derived insulation materials can move from experimental demonstration toward robust deployment in building applications. This shift from simple waste encapsulation to high-value material hybridization represents a viable and opportune pathway to meet both stringent building codes and ambitious net-zero targets.

## Figures and Tables

**Table 1:** Comparative properties of textile fiber-based composites.

Type	Textile Fiber	Other Materials (Reinforcement/Aggregate/Skeleton)	Binder/Matrix/Adhesive	Textile Waste Type	Thermal Conductivity (W/(m·K))	Density (kg/m^3^)	Compressive Strength (MPa)	Flexural Strength (MPa)	Noise Reduction Coefficient (NRC)	Ref.
Natural fibers	Sheep wool	Cellulose; rPET fibers; rPES fibers; hemp fibers.	Carding-folding; PET; Co-PET/PET; chitosan; arabic gum; polylactic acid (PLA).	Post-industrial	0.030–0.060	N/A	0.10–0.20	N/A	0.40–0.65	[8,33,40,41,42,43,44]
	Jute	Coffee husk post-consumption; paper cups; coffee grounds.	Hydrothermal compression.	Post-consumer	0.038–0.044	670–920	N/A	N/A	N/A	[45]
	Flax	Fiber glass; ash; sand; coconut fiber; jute felt; hemp felt; linen felt; cork.	Arabic natural glue; polyester; PLA; epoxy resin.	Pre-consumer	0.029–1.177	240–340	N/A	2.1–3.35	0.15–0.20	[13,46,47,48,49,50]
	Cotton	Hemp particules (hurd); coir pith.	Mycelium; epoxy; calcium alginate; gypsum.	Pre-consumer	0.029–1.173	72–730	0.28–1.57	0.06–3.35	0.15–0.83	[11,16,51,52,53,54]
	Hemp	Glass fibers; expanded polystyrene (EPS); rapeseed straw.	Cement mortar; cement milk; corn starch; calcium sulfate hemihydrate (gypsum).	Virgin fibers or raw agricultural material	0.041–0.089	21–1320	N/A	2.1–4.1	N/A	[55,56,57,58]
	Flax and hemp	Wood fiber; miscanthus.	Sodium silicate; polyethylene fibers; polypropylene.	Virgin textile fibers	0.040–0.047	56–83	N/A	N/A	N/A	[12]
Natural and synthetic fibers	Textile Mix and hemp	Wood; mixed fibers.	Polyester; polyolefins.	Pre-consumer, Post-industrial	N/A	N/A	N/A	N/A	N/A	[59]
	Sheep wool and polypropylene fabric	Tetra Pak; glass fiber; jute.	Polypropylene spunbonded nonwoven fabric.	Pre-consumer, post-consumer, and virgin fibers	0.055–0.062	N/A	N/A	N/A	N/A	[14]
	Cotton/polyester	N/A	Natural rubber.	post-industrial	N/A	N/A	N/A	N/A	0.5–0.7	[60]
	Wool with cotton and polyester with nylon	N/A	N/A	Post-consumer	0.039–0.049	20–50	N/A	N/A	N/A	[61]
	Cotton, flax, and polyester	N/A	Portland cement.	Pre-consumer and post-consumer	0.830–1.170	1600–1900	N/A	N/A	0.2–0.5	[9]
	Flax and textile waste	Jute; hemp; coconut fiber.	Biodegradable epoxy resin; polyester.	Pre-consumer and virgin fibers	N/A	1010–1230	N/A	N/A	N/A	[62]
Mixed textile waste fibers	Mixed textile waste	Residual wood sawdust; olive waste; grass cuttings; dry leaves; sugarcane bagasse; polypropylene carpet fibers; palm oil fuel ash; glass wool; cotton stalk fiber.	Kelp brown algae; bivalve mollusc shells; synthetic fiber; unsaturated polyester resin; acrylic adhesive; white cement; Paris plaster; sand; polypropylene textile.	Pre-consumer, post-industrial, and post-consumer	0.85–1.04	68–1850	1.54–3.11	0.05–2.62	0.18–0.46	[63,64,65,66,67]
Synthetic fibers	Denim	Miscanthus; rice husk; wheat husk; wood fibers.	Corn starch; chitosan; glacial acetic acid; polibutileno adipato-co-tereftalato (PBAT); PLA; sodium alginate;	Post-industrial and post-consumer	0.076–1.140	30–488	0.20–11	0.21–1.65	N/A	[68,69,70,71]
	Polyester	Expanded polystyrene; end-of-life tires (ELT).	Polyvinyl acetate (PVAc); plaster (Gypsum).	Post-industrial	0.050–0.210	759–1390	4.4–8.5	1–13	0.15–0.59	[72,73]

**Table 2:** Comparative properties of fiberglass-based composites: thermal, mechanical, acoustic performance, and recyclability.

Material	Other Materials (Reinforcement/Aggregate/Skeleton)	Binder/Matrix/Adhesive	Fiberglass Recycled?	Thermal Conductivity (W/(m·K))	Density (kg/m^3^)	Compressive Strength (MPa)	Flexural Strength (MPa)	Noise Reduction Coefficient (NRC)	Ref.
Fiberglass	Natural fibers	Composite Matrix/Binder Not Specified	X	N/A	N/A	N/A	N/A	N/A	[77]
	FRP grid/Fibrous mesh grid	Mortar/Geopolymer	✓	0.234–0.83	N/A	5.9–9.1	2.2–3.1	N/A	[78]
	Waste tire rubber	Inorganic Matrix Mortar	X	0.030–0.033	N/A	1.87–6.86	0.36–1.85	N/A	[79]
	Crushed brick aggregate/Crushed limestone aggregate/Basalt fiber	White Portland Cement	✓	N/A	467–1807	0.92–8.54	0.59–3.06	0.23–0.34	[80]
	Calamus rotang (Rattan) fiber/powder	Epoxy	X	N/A	N/A	N/A	96–181	N/A	[81]
	Snail shell particulates (Bio-derived calcium carbonate)	Recycled Polypropylene RPP	X	0.120–0.380	N/A	N/A	30.16–34.56	N/A	[82]
	Tetra Pak waste/Jute fabric/Wool fiber waste	Polypropylene Nonwoven Fabric/PP	X	0.055–0.062	N/A	N/A	N/A	N/A	[14]
	Hemp fibers	Gypsum	X	N/A	N/A	N/A	N/A	N/A	[55]
	Gypsum/Tissue paper	Gypsum Matrix/Natural Rubber Latex	X	0.063–0.338	712.5–1136.3	0.19–1.98	0.009–0.900	0.13–0.39	[7]
	Flax fiber	Polylactic Acid PLA	X	N/A	1240–1450	N/A	N/A	N/A	[49]
		Bicomponent Polyester Fiber	X	0.030–0.054	210–400	N/A	0.6–3.5	N/A	[83]
	Date palm fiber (DPF)	Unsaturated Polyester Resin	X	0.214–0.231	1582–1900	N/A	14.71–105.71	N/A	[84]
		ABS Resin/SAN	X	0.17–0.22	N/A	N/A	N/A	N/A	[85]
	Centrifugal glass wool	Gypsum Matrix	X	N/A	N/A	N/A	N/A	0.77	[86]
	Flax fiber/Agglomerated cork	Epoxy Resin	X	N/A	240–340	N/A	N/A	0.15–0.18	[13]
	Polyester resin/PVC sheets	Polyester Resin	✓	0.133–0.190	445.5–604.2	N/A	N/A	N/A	[10]
	Ultrafine glass micro/nano fibers	Polyimide PI Resin	X	N/A	N/A	N/A	N/A	0.60–0.70	[87]

**Table 3:** Solutions using bio-binders in composition and formulation.

Material	Bio-Binder	Thermal Conductivity (W/(m·K))	Density (kg/m^3^)	Compressive Strength (MPa)	Flexural Strength (MPa)	NRC	Characterization and Properties	Year	Ref.
Flax fiber waste	Arabic natural glue	0.029–0.325	25–650	N/A	N/A	N/A	Modified guarded hot box; steady state	2025	[50]
Cotton and viscose waste fibers	Calcium alginate	N/A	N/A	N/A	N/A	N/A	Flammability, DSC/DTA-TG, cone calorimetry, SEM, EDS, XRD, STA-FTIR-GC/MS, TGA	2023	[51]
Denim textile waste	Corn starch	N/A	30–40	N/A	N/A	N/A	SEM, viscous properties, acoustic properties, surface hardness, and MOR	2022	[71]
Beech sawdust and basalt fibers	Polylactic acid (PLA)	0.080–0.090	1140–1570	N/A	28–79	N/A	water absorption, tensile test, flexural test, Impact strength, STL, *λ*, TGA, FTIR, SEM	2022	[89]
Bamboo particles	Mycelium	0.080	229	N/A	N/A	N/A	Hot Disk, LCA, and *λ*	2022	[90]
Cotton/polyester mixed waste	Natural rubber latex	N/A	N/A	N/A	N/A	0.65–0.80	SAC, NCR, *E*	2021	[60]
Miscanthus × giganteus, rice husk, Textile wastes	Chitosan	0.076–0.084	334–405	0.2–0.64	0.48–0.45	N/A	*ρ*_bulk_, *ρ*_true_, *N*, *λ*, flexural test, compression test, and SEM	2021	[70]
Jute fiber, biaxial linen fiber, coconut fiber, hemp fiber, and textile fiber waste	Epoxy resin from Karanja oil	N/A	1030–1180	N/A	N/A	N/A	SEM, Vibration test, Acoustic transmission	2020	[62]
Merino wool fibers	Gum arabic and chitosan	0.049–0.060	80–197	N/A	N/A	N/A	SEM, *N*, SAC, *ρ*_true_, *T*, σ, Ignitability, JCA, and *λ*	2019	[42]
Merino wool fibers	Chitosan	N/A	80–197	N/A	N/A	N/A	σ, *ρ*_true_, *T*, *N*	2019	[40]
Wood fibers and textile waste	Sodium alginate	0.078–0.089	308–333	0.44–1.41	0.20–0.54	N/A	Gel time, crosslinking, thermal stability, DSC, *λ*	2018	[68]
Rape straw and hemp straw	Corn starch	0.041–0.089	64–140	N/A	N/A	N/A	*λ*	2017	[57]
Hemp fibers and sheep wool waste	Polylactic acid (PLA)	0.034–0.045	40	N/A	N/A	0.60–0.65	SEM, *λ*, SAC and NRC	2017	[33]

## Data Availability

Data created and analyzed in this study are available upon request.

## Abbreviations and Used Symbols

The following abbreviations and symbols are used in this manuscript:

DSC	Differential scanning calorimetry
*E*	Modulus of elasticity, MPa
EDS	Energy dispersive spectroscopy
FRP	Fiber-reinforced polymer
FTIR	Fourier transform infrared spectroscopy
JAC	Obtained using the Johnson–Champoux–Allard method
MOR	Modulus of rupture
*N*	Open porosity
NP	Number of publications
NRC	Noise reduction coefficient
PET	Polyethylene terephthalate
PY_start	Year of first publication
SAC	Sound absorption coefficient
SEM	Scanning electron microscopy
STL	Sound transmission loss
*T*	Tortuosity
TC	Total citations
TGA	Thermogravimetric analysis
TPS	Transient plane source
XRD	X-ray diffraction analysis
*λ*	Thermal conductivity, W/(m·K)
*ρ*_bulk_	Bulk density, kg/m^3^
*ρ*_true_	True density, kg/m^3^
*σ*	Air flow resistivity, kN·s/m^4^
